# Associations of gender identity with sexual behaviours, social stigma and sexually transmitted infections among adults who have sex with men in Abuja and Lagos, Nigeria

**DOI:** 10.1002/jia2.25956

**Published:** 2022-07-06

**Authors:** Elyse LeeVan, Fengming Hu, Andrew B. Mitchell, Afoke Kokogho, Sylvia Adebajo, Eric C. Garges, Haoyu Qian, Julie A. Ake, Merlin L. Robb, Manhattan E. Charurat, Stefan D. Baral, Rebecca G. Nowak, Trevor A. Crowell, for the TRUST/RV368 Study Group

**Affiliations:** ^1^ U.S. Military HIV Research Program Walter Reed Army Institute of Research Silver Spring Maryland USA; ^2^ Henry M. Jackson Foundation for the Advancement of Military Medicine Bethesda Maryland USA; ^3^ Institute of Human Virology University of Maryland Baltimore Maryland USA; ^4^ HJF Medical Research International Abuja Nigeria; ^5^ Center for International Health and Biosecurity (Ciheb) Abuja Nigeria; ^6^ Department of Preventive Medicine and Biostatics Uniformed Services University Bethesda Maryland USA; ^7^ Johns Hopkins Bloomberg School of Public Health Baltimore Maryland USA

**Keywords:** social stigma, sexual and gender minorities, transgender persons, gonorrhoea, chlamydia, Africa South of the Sahara

## Abstract

**Introduction:**

Sexual and gender minority populations are disproportionately affected by the global syndemic of HIV and other sexually transmitted infections (STIs). We hypothesized that transgender women (TGW) and non‐binary individuals in Nigeria have more STIs than cis‐gender men who have sex with men (cis‐MSM), and that experiences of stigma and sexual practices differ between these three groups.

**Methods:**

From 2013 to 2020, TRUST/RV368 enrolled adults assigned male sex at birth who reported anal sex with men in Abuja and Lagos, Nigeria. Participants were tested for STIs and completed questionnaires about sexual behaviours and social stigma every 3 months. Participants were categorized as cis‐MSM, TGW or non‐binary/other based on self‐reported gender identity. Gender group comparisons were made of HIV, gonorrhoea and chlamydia prevalence and incidence; stigma indicators; and condom use during anal sex.

**Results:**

Among 2795 participants, there were 2260 (80.8%) cis‐MSM, 284 (10.2%) TGW and 251 (9.0%) non‐binary/other individuals with median age of 23 years (interquartile range 20–27). HIV prevalence among cis‐MSM, TGW and non‐binary/other participants was 40.8%, 51.5% and 47.6%, respectively (*p* = 0.002). HIV incidence was 8.7 cases per 100 person‐years (PY) (95% confidence interval *[CI] 6.9–10.8)*, 13.1 cases/100 PY (95% CI 6.5–23.4) and 17.6 cases/100 PY (95% CI 9.8–29.0, *p* = 0.025), respectively. Anorectal gonorrhoea incidence was lower in cis‐MSM than TGW (22.2 [95% CI 19.6–25.0] vs. 35.9 [95% CI 27.3–46.3]). TGW were more likely than cis‐MSM to report being affected by stigma, including assault (47.2% vs. 32.3%), fear of walking around (32.4% vs. 19.2%) and healthcare avoidance (25.0% vs. 19.1%; all *p* < 0.05). TGW were more likely to report always using condoms than non‐binary/other individuals (35.3% vs. 26.2%, *p* = 0.041) during receptive anal sex.

**Conclusions:**

Sexual and gender minorities in Nigeria have heterogeneous sexual behaviours and experiences of social stigma that may influence the vulnerability to HIV and other STIs. There is a need for tailored interventions that acknowledge and are informed by gender. Further research is needed, particularly among understudied non‐binary individuals, to better understand disparities and inform tailored interventions to improve outcomes among these communities.

## 1 INTRODUCTION

At least 25% of the global burden of HIV is borne by individuals who are sexual or gender minorities, which include individuals who identify as lesbian, gay, bisexual, asexual, transgender or queer; individuals with same‐sex or same‐gender attractions or behaviours; individuals with a difference in sex development; and other individuals with non‐binary constructs of sexual orientation, gender identity or expression, or reproductive development [1, 2]. Prior studies have also demonstrated increased prevalence of other sexually transmitted infections (STIs) in these groups [3, 4, 5, 6]. Increased risk of HIV and other STIs may be driven by the efficiency of HIV transmission during condomless anal sex, impeded healthcare access due to discrimination and stigma, provider unawareness of unique healthcare needs and socio‐cultural marginalization [7, 8, 9, 10, 11, 12, 13]. There is a lack of data‐driven recommendations regarding delivery of sensitive and appropriate preventive care and treatment [6, 14], particularly in regions where sexual and gender minority communities are stigmatized and HIV is prevalent, such as sub‐Saharan Africa [15, 16, 17].

There is an unmet need to elucidate risk factors and effective interventions to improve health outcomes among key populations, such as men who have sex with men (MSM), transgender individuals and gender non‐binary individuals [18]. These individuals are often grouped together under the LGBTQ heading, potentially obscuring specific healthcare needs and other differences among its member groups [19, 20, 21, 22, 23]. For example, an analysis from eight African countries revealed that transgender women (TGW) had higher HIV prevalence than cisgender MSM (cis‐MSM) and were more likely to report condomless receptive anal sex, rape and exclusion from family gatherings [24].

Nigeria, Africa's most populous country, has institutional and cultural stigmatization of sexual and gender minority populations, including criminalization of same‐sex sexual practices [25]. HIV prevalence in Nigerian individuals aged 15–65 is approximately 1.9% overall, but national data from 2014 suggest that HIV prevalence in MSM was 22.9%, higher than any other key population and representing a sharp increase from 13.5% in 2007 [25, 26, 27, 28, 29]. Early data from the TRUST/RV368 study of sexual and gender minority populations attending trusted community‐based clinics in Abuja and Lagos, Nigeria, showed that more than half of participants were living with HIV at enrolment [29]. Gonorrhoea and chlamydia prevalence were also higher than contemporary global assessments among reproductive aged adults [29, 30, 31].

In previous analyses from TRUST/RV368, significant differences were identified in HIV incidence and prevalence between cis‐MSM and TGW [7, 29, 30, 32], but early analyses were not adequately powered to fully explore associations between gender identity and key clinical outcomes. We hypothesized that TGW and non‐binary individuals in Nigeria have more STIs than cis‐MSM and that stigma and sexual practices differ between these three groups. We used updated data to perform a comprehensive analysis addressing the research question of how STIs, sexual behaviours and stigma differ between groups with different gender identities. Understanding gender‐related differences that influence HIV and STI vulnerability may inform targets for enhancing tailored delivery of HIV prevention and treatment to maximize efficacy.

## 2 METHODS

### 2.1 Study population

The TRUST/RV368 study was a prospective, observational cohort that enrolled sexual and gender minority participants at specialized community health centres in Abuja and Lagos, Nigeria, using respondent‐driven sampling (RDS) as previously described [29, 33, 34, 35]. Each of 12 seeds recruited up to 33 waves with equilibrium reached after 14 waves. Previously published diagnostic analyses suggested that the study population was a representative cross‐section of the SGM population in each city [36]. Participants aged at least 16 years in Abuja or 18 years in Lagos were eligible for enrolment if they were assigned male sex at birth, reported anal intercourse with a male partner in the preceding year and presented a valid RDS coupon. Participants completed enrolment procedures at two visits approximately 2 weeks apart, including a comprehensive demographic and behavioural questionnaire as well as testing for HIV and other STIs. Testing and questionnaire modules were repeated every 3 months thereafter. The study opened for enrolment in Abuja in 2013 and in Lagos in 2014. The study initially included a total of seven follow‐up visits over 18 months but was extended for active participants at the Abuja site in 2017 to up to 13 follow‐up visits over 36 months. Follow‐up in Lagos ended in 2018 and data from ongoing follow‐up in Abuja were censored in October 2020 for these analyses.

### 2.2 Definition of gender and sexual orientation

A validated, two‐step gender assessment was used for this study [37]. All participants were assigned male sex at birth. At enrolment, participants were asked, “What do you consider your gender to be?” Response options included: man, woman, other (specify), both male and female, refusal and don't know. For these analyses, participants who selected “man” were considered cisgender men, those who selected “woman” were considered TGW and participants who selected “other,” “both male and female,” “don't know,” or “refuse to answer” were categorized as non‐binary/other. Sexual orientation was ascertained by asking “What do you consider your sexual orientation to be?” Responses included: gay or homosexual, bisexual, queer, heterosexual or straight, other, refusal and don't know.

### 2.3 Sexual behaviours and condom use

Sexual behaviours were assessed via a structured interview conducted by trained study staff. At enrolment, this interview included questions about sexual behaviours with men in the preceding 12 months. Participants who reported a specific sexual activity, such as insertive anal sex, were then asked how often condoms were used. Participants who answered “always” were categorized as having consistent condom use, while all other responses were considered less than consistent, recognizing that consistent condom use reduces HIV transmission [38, 39, 40].

### 2.4 Testing for HIV, gonorrhoea and chlamydia

Participants were tested for HIV at enrolment and every 3 months thereafter with fingerstick blood specimens utilizing a parallel algorithm of Determine® (Alere, Waltham, MA, USA) and Uni‐gold® (Trinity Biotech, Country Wicklow, Ireland) [7, 29, 35]. Participants were provided pre‐ and post‐test counselling at each study visit and were offered immediate antiretroviral therapy for HIV, if diagnosed.

The local standard of care during the study relied on syndromic diagnosis of STIs, but study participation included universal screening for STIs every 3 months at up to three anatomic sites. Urine and anorectal swab specimens were used for real‐time PCR‐based testing for gonorrhoea and chlamydia every 3 months using the Aptima Combo® assay (Hologic, Bedford, MA, USA) [7, 29, 35, 41]. Oropharyngeal swabs were also tested every 3 months from October 2014 through January 2019. Bacterial STIs were treated with antibiotics according to local guidelines. Other care enhancements offered to participants included counselling on STI prevention, access to free condoms and condom‐compatible lubricants, and referral for pre‐exposure prophylaxis (PrEP) once regionally available.

### 2.5 Stigma

At enrolment, participants were asked to report their lifetime experiences of stigma in the private, societal and healthcare settings using a tool validated in a population with similar geography, gender identification and sexual practices [42]. Stigma subcategories included perceived, experienced and anticipated stigmas [7, 13]. Some questions solicited stigma indicators without attribution as to the reason, such as sexual violence (“Have you ever been forced to have sex when you did not want to?[By forced, I mean physically forced, coerced to have sex, or penetrated with an object when you did not want to]”), and assault (“Have you ever been pushed, shoved, slapped, hit, kicked, choked, or otherwise physically hurt by someone?”). Other questions specifically asked about stigma “because you have sex with men,” including questions about blackmail, fear of walking around, denial of healthcare, avoidance of healthcare and fear of seeking healthcare.

### 2.6 Statistical analyses

Demographic characteristics at enrolment were summarized using means, medians with interquartile range and frequencies. Demographics were compared between gender groups using Pearson's Chi‐squared. HIV prevalence was calculated by dividing the number of cases at enrolment by the total number of participants tested and multiplying by 100 to yield a percentage. Prevalence of gonorrhoea and chlamydia was calculated at the time of first test, with separate calculations for each anatomic site (urogenital, anorectal and oropharyngeal). Pairwise comparisons of prevalence by gender group were made using the Tukey method.

Incidence rates were calculated by dividing the total number of cases by the population observation time at risk and multiplying by 100 to yield cases per 100 person‐years (PY). Participants with prevalent HIV contributed no observation time to incidence analyses. Participants with prevalent or repeated infections with gonorrhoea or chlamydia contributed observation time after documented clearance with a negative PCR. Incidence rate ratios with 95% confidence intervals were used to test for differences between gender groups. Among participants without HIV, Kaplan–Meier curves were generated to depict the time to incident infection, by gender, with global comparisons using log‐rank tests. Pairwise comparisons applied the Sidak correction to adjust *p* values. The prevalence of stigma indicators and condom use at enrolment were compared between gender groups using the two‐sample test of proportions.

For all analyses, statistical significance was defined as *p*<0.05. Analyses were performed using Stata 15.0 (StataCorp LP, College Station, TX, USA) and SAS 9.4 (SAS Institute Inc, Cary, NC, USA).

### 2.7 Ethical approval

All participants provided written informed consent before enrolment. The study was approved by institutional review boards at the Walter Reed Army Institute of Research, Silver Spring, MD, USA; the University of Maryland, Baltimore, MD, USA; and the National Health Research Ethics Committee and Nigerian Ministry of Defense Health Research Ethics Committee, Abuja, Nigeria.

## 3 RESULTS

### 3.1 Demographics

In total, 2795 participants were enrolled, including 2260 (80.8%) cis‐MSM, 284 (10.2%) TGW and 251 (9.0%) non‐binary/other individuals. The non‐binary/other group included 215 participants who characterized their gender as “both male & female,” 18 “versatile,” 8 “don't know,” 5 “other” without specification, 2 “refused to answer,” and 3 with missing data. Gender groups differed by age, research site distribution and sexual orientation (Table 1).

**Table 1:** Demographics by gender

Characteristic	Cisgender MSM (*N* = 2260)	Transgender women (*N* = 284)	Non‐binary/other^a^ (*N* = 251)	*p*
Age				**0.01**
≤21	742 (32.8%)	110 (38.7)	84 (33.5%)
22–30	1275 (56.4%)	156 (54.9%)	139 (55.4%)
>30	243 (10.8%)	18 (6.3%)	28 (11.2%)
Site, *n* (%)				
Abuja	1744 (77.2%)	184 (64.8%)	195 (77.7%)	**<0.001**
Lagos	516 (22.8%)	100 (35.2%)	56 (22.3%)	
Education level				0.054
Junior secondary or less	300 (13.3%)	38 (13.4%)	21 (8.4%)	
Senior secondary	1168 (51.7%)	167 (58.8%)	145 (57.8%)	
Higher than senior secondary	787 (34.8%)	78 (27.5%)	84 (33.5%)	
Unknown	5 (0.2%)	1 (0.4%)	1 (0.4%)	
Marital status				0.15
Single/never married	2041 (90.3%)	261 (91.9%)	236 (94.0%)	
Married/living with a woman	146 (6.5%)	10 (3.5%)	8 (3.2%)	
Living with a man	22 (1.0%)	4 (1.4%)	3 (1.2%)	
Divorce/separated/widowed/other	51 (2.3%)	9 (3.2%)	4 (1.6%)	
Sexual orientation				**<0.001**
Gay/homosexual	654 (28.9%)	161 (56.7%)	86 (34.3%)	
Bisexual	1599 (70.8%)	121 (42.6%)	162 (64.5%)	
Other/missing^b^	7 (0.3%)	2 (0.7%)	3 (1.2%)	

Note: Data are presented as *n* (%). *p* Values were calculated using a Pearson's Chi‐squared test. Statistically significant *p* values (<0.05) are in bold.

^a^ In the non‐binary/other category, responses included: both male and female = 215, versatile = 18, refusal = 2, don't know = 8, other (unspecified) = 5, missing = 3.

^b^ The other category includes two cis‐MSM who identified as “heterosexual,” one cis‐MSM who identified as “queer,” three cis‐MSM who replied “don't know,” one cis‐MSM with missing data, two TGW who identified as “transgender,” one non‐binary/other individual who refused to answer and two non‐binary/other individuals with missing data.

### 3.2 HIV and other STIs

**Figure 1:**
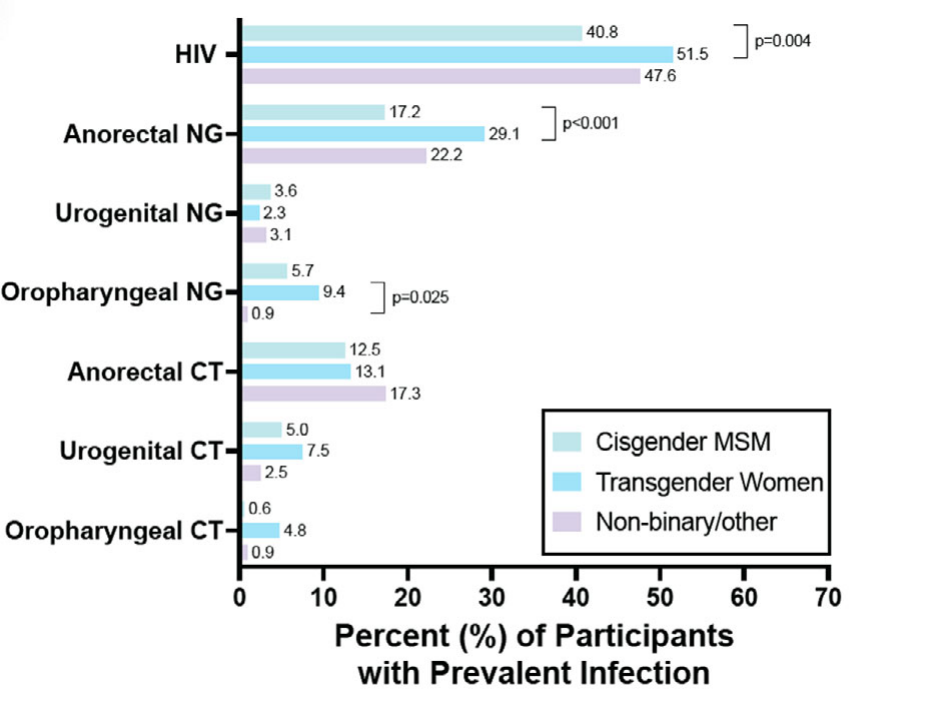
STI prevalence among study participants stratified by participant gender. Pairwise comparisons between gender groups were performed by a Tukey test and statistically significant *p* values (≤0.05) are presented.

Overall HIV prevalence was 42.5%, including 37.2% in Abuja and 58.2% in Lagos. Among cis‐MSM, TGW and non‐binary/other participants, HIV **prevalence** was 40.8%, 51.5% and 47.6%, respectively (*p* = 0.002; Figure 1). As compared to cis‐MSM, TGW had a higher prevalence of HIV (51.5% vs. 40.8%, *p* = 0.004) and anorectal gonorrhoea (29.1% vs. 17.2%, *p*<0.001). TGW also had a higher prevalence of oropharyngeal gonorrhoea than non‐binary/other individuals (9.4% vs. 0.9%, *p* = 0.03). There were no differences in urogenital gonorrhoea or site‐specific chlamydia prevalence between gender groups.

**Figure 2:**
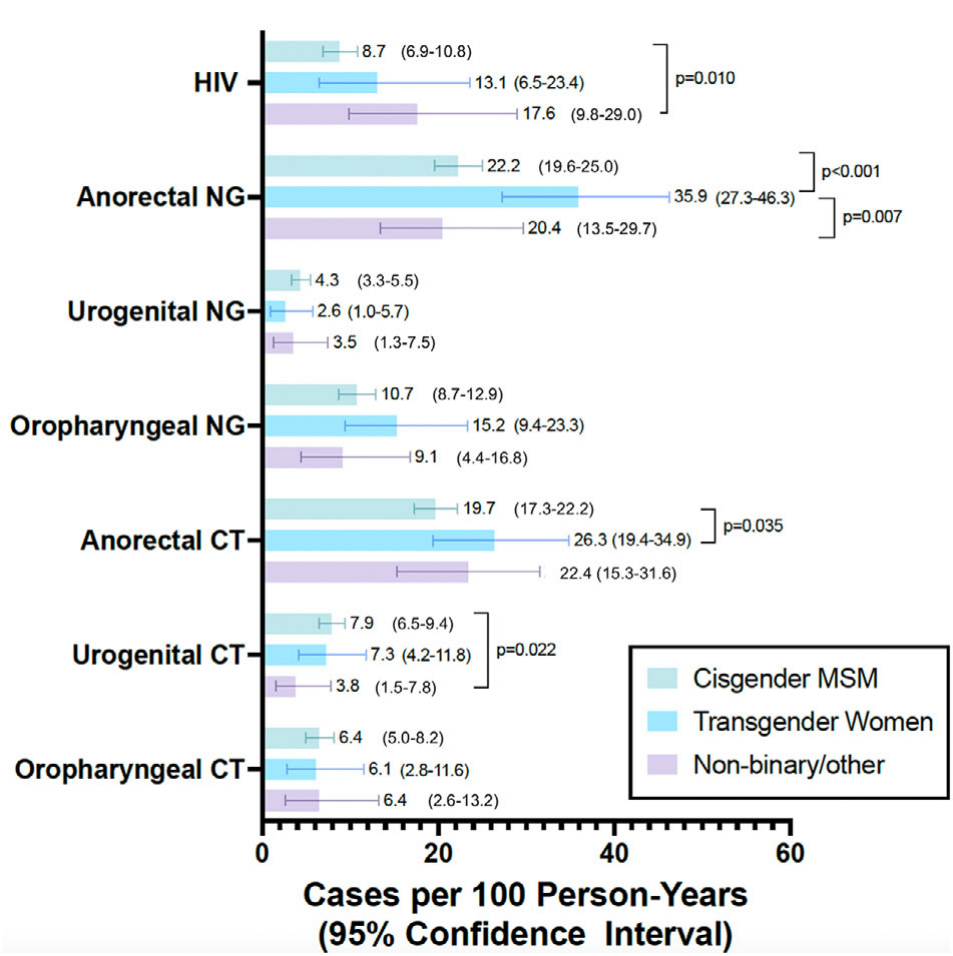
STI incidence among study participants stratified by participant gender. Pairwise comparisons of incidence rate ratios between gender groups were performed and statistically significant *p* values (≤0.05) are presented.

Among cis‐MSM, TGW and non‐binary other participants, HIV incidence was 8.7/100 PY (95% CI 6.9–10.8), 13.1 cases/100 PY (95% CI 6.5–23.4) and 17.6 cases/100 PY (95% CI 9.8–29.0), respectively (*p* = 0.025; Figure 2). HIV incidence was significantly higher among non‐binary/other participants as compared to cis‐MSM (*p* = 0.010).

The incidence of anorectal gonorrhoea among TGW was 35.9 cases/100 PY (95% CI 27.3–46.3), which was significantly higher than among cis‐MSM (22.2 cases/100 PY, 95% CI 19.6–25.0, *p*<0.001) and non‐binary/other participants (20.4 cases/100 PY, 95% CI 13.5–29.7, *p*<0.001). The incidence of anorectal chlamydia was higher among TGW than cis‐MSM participants (26.3 cases/100 PY [95% CI 19.4–34.9] vs. 19.7 cases/100 PY [95% CI 17.3–22.2], *p* = 0.035). The incidence of urogenital chlamydia was higher among cis‐MSM than non‐binary/other participants (7.9 cases/100 PY [95% CI 6.5–9.4] vs. 3.8 cases/100 PY [95% CI 1.5–7.8], *p* = 0.022).

**Figure 3:**
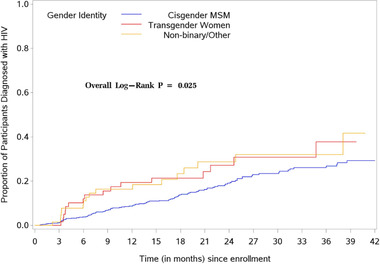
Time to event analyses for HIV from enrolment. Only individuals with an initial negative HIV test were included. The x‐axis was truncated at 42 months because very few participants were followed beyond that time.

In time‐to‐event analyses, there were differences between the three gender groups in the time from enrolment to incident HIV (*p* = 0.025; Figure 3). TGW had a shorter time to first incident anorectal gonorrhoea infection than other gender groups (log‐rank *p* = 0.026), but there were no significant differences in time to first urogenital (log‐rank *p* = 0.419) or oropharyngeal (log‐rank *p* = 0.204) gonorrhoea infection. There were no significant differences by gender group in time to first chlamydia infection at the anorectal (log‐rank *p* = 0.21), urogenital (log‐rank *p* = 0.23) or oropharyngeal (log‐rank *p* = 0.98) sites.

### 3.3 Stigma

**Figure 4:**
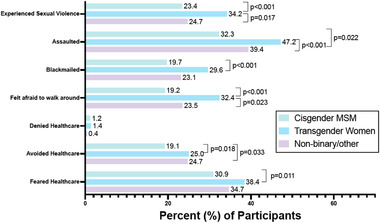
Bars represent the percentage of participants (by gender) who reported each indicator of stigma at the time of enrolment. Pairwise comparisons are made between gender groups for each stigma indicator, with significant *p* values (≤0.05) presented. Questionnaire data, including blackmailed, felt afraid to walk around, denied healthcare, avoided healthcare and feared healthcare, were specifically collected in reference to a participant's status as an MSM.

TGW were more likely than cis‐MSM to report almost all stigma indicators, including sexual violence, assault, blackmail, fear of walking around, avoidance of healthcare and fear of seeking healthcare (Figure 4). TGW were also more likely than non‐binary individuals to report fear of walking around and experience of sexual violence. Non‐binary individuals were more likely to report healthcare avoidance and assault than cis‐MSM.

### 3.4 Sexual behaviours and condom use

**Figure 5:**
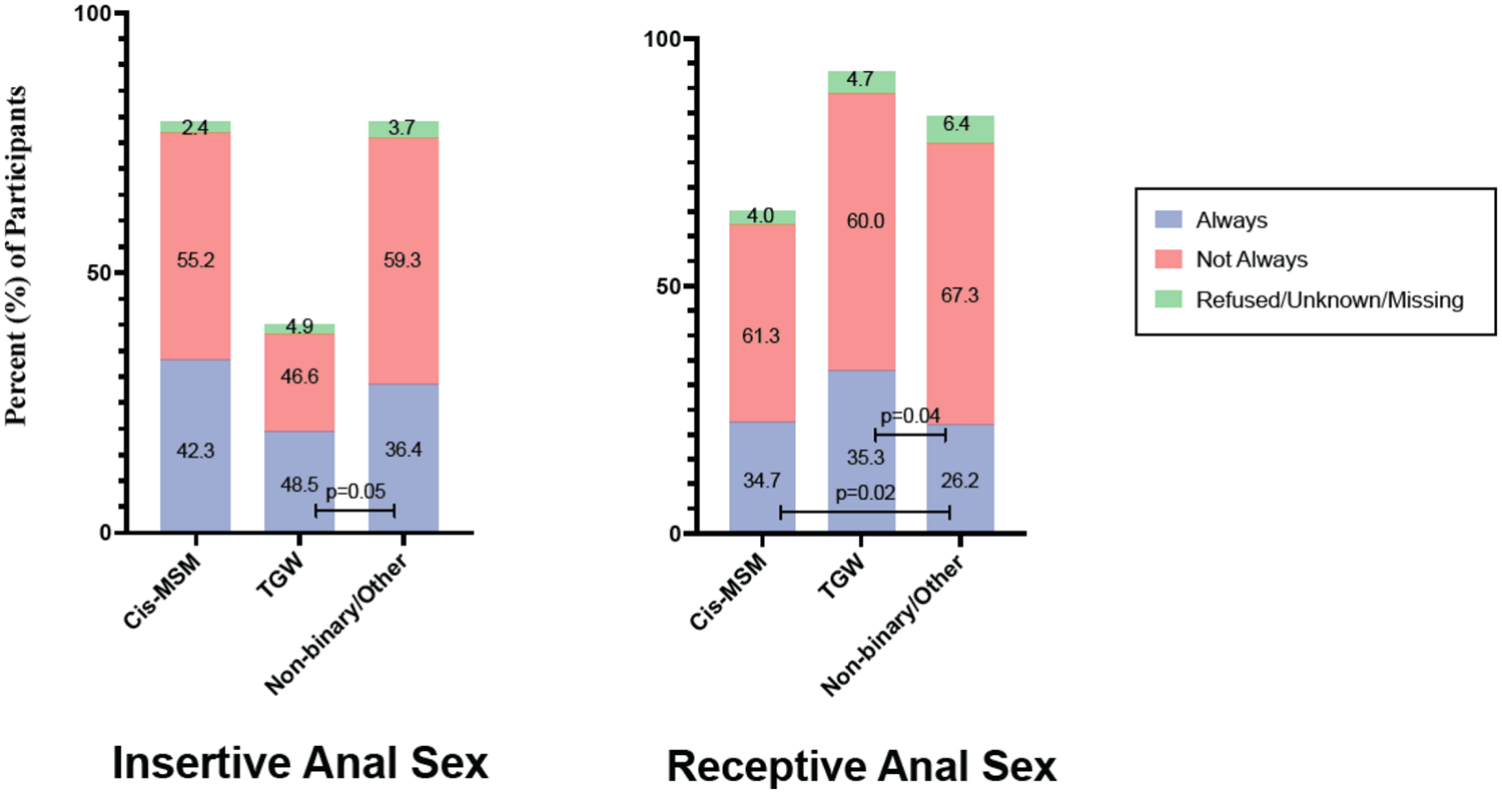
Condom use by gender. The bar height represents the percentage of participants of each gender who reported insertive (left) or receptive (right) anal sex within the 12 months prior to enrolment. The colour bars represent the percent of participants who reported each frequency of condom use in the cis‐MSM, TGW and non‐binary/other gender groups. A two‐sample test of proportions was used to compare each response type between gender groups. Statistically significant *p* values (≤0.05) are presented.

Behavioural differences were noted between gender groups (Figure 5). TGW were more likely to report no participation in insertive anal sex in the last year (54.2%) than cis‐MSM (19.0%), or non‐binary/other people (19.9%, *p*<0.001 for both comparisons). Cis‐MSM were more likely to report no participation in receptive anal sex in the last year (31.8%) than TGW (6.0%), or non‐binary/other people (15.1%, *p*<0.001 for both comparisons). Non‐binary/other individuals were also more likely to report no participation in receptive anal sex than TGW (15.1% vs. 6%, *p*<0.001).

Of participants who reported participating in insertive anal sex, TGW were more likely than non‐binary/other individuals to report always using condoms (48.5% vs. 36.4%, *p* = 0.045). Of participants who reported receptive anal sex, both cis‐MSM and TGW were more likely to report always using condoms than non‐binary/other individuals (34.7% vs. 26.2%, *p* = 0.017 and 35.3% vs. 26.2%, *p* = 0.041).

## 4 DISCUSSION

We found a high overall burden of HIV and other STIs among Nigerian sexual and gender minority populations. TGW had particular high prevalence and incidence of HIV, which is consistent with previous literature [14, 43, 44]. Prior data on non‐binary individuals are generally lacking or grouped with transgender populations, but we found that almost half of non‐binary participants in our study had HIV at enrolment, which was lower than TGW in our study, but higher than the HIV prevalence reported for transgender populations internationally [45]. HIV incidence among non‐binary individuals in our study was significantly higher than that observed among cis‐MSM and numerically higher than TGW, though the latter difference was not statistically significant. Regional and international STI clinical guidelines, including the World Health Organization Global Strategies, increasingly define MSM and transgender people as key populations at risk for HIV and other STIs, applying evidence‐informed clinical care metrics, but do not mention non‐binary populations [46, 47]. The paucity of data in non‐binary populations limits the ability to define similar population‐based risk reduction measures.

There is a well‐recognized need for data regarding the syndemic of HIV and other STIs, particularly among sexual and gender minorities [6, 48]. Consistent with prior studies, we found that anorectal gonorrhoea and chlamydia were common among MSM [49]. Furthermore, our data reinforce recent reports of higher prevalence and incidence of STIs in TGW compared to cisgender men and higher rates of extra‐genital infections [6, 50, 51]. The high burden of gonorrhoea and chlamydia at the anorectal site for TGW may be attributable to distinct sexual practices, such as a higher likelihood of receptive anal intercourse as compared to cis‐MSM in our cohort. Sexual networks are also known to impact STI transmission geometry in MSM [52]. Differing compositions of sexual networks and sexual partnership dynamics between cis‐MSM and TGW have been identified [53, 54], which may drive differences in disease prevalence between these groups. Additional, focused, studies are required to better understand the sexual practices, identities and networks of non‐binary individuals, including qualitative research.

The combinations of identities and orientations that were seen in our study demonstrate interesting relationships that are likely contributory to STI vulnerability. The majority of TGW in this study self‐identified as gay/homosexual, while the majority of cis‐MSM and non‐binary/other individuals self‐identified as bisexual. Although heterosexual was provided as a response option, only two participants identified with this sexual orientation. We did not ask about gender preferences for sexual partners, but recent data have suggested that birth males who are sexually attracted exclusively to men have heightened HIV risk, even when compared to other men and TGW who have sex with men but are sexually attracted to women [55].

Despite reporting a higher frequency of consistent condom use than other gender groups, TGW had increased incidence of anorectal STIs. Although condom use was more consistent than in other groups, it may have been insufficient to reduce transmission driven by higher engagement in anal receptive sex. By extension, this may also suggest that condom‐related messaging alone is unlikely to be sufficient to curb HIV transmission, but must be accompanied by biologic interventions, such as PrEP with demonstrated safety and efficacy in sexual and gender minority populations [56, 57]. Further studies are needed to address PrEP efficacy and uptake in TGW. The iPrEx trial subgroup analysis of TGW demonstrated decreased PrEP efficacy compared to cis‐MSM, likely due to decreased regimen adherence, but other studies have shown conflicting results [58]. Decreased condom use in the non‐binary compared to TGW and cis‐MSM populations in our study suggests a potential opportunity for condom‐promoting interventions.

The high prevalence and incidence of gonorrhoea and chlamydia at the anorectal site merits further investigation of extragenital routes of transmission. Implementation of routine STI screening of extragenital sites for sexual and gender minority populations is recommended as extragenital STIs are less likely to be symptomatic, yet more common than urethral STIs in these populations [44]. Providing STI PrEP and post‐exposure prophylaxis could be explored as biologic interventions to prevent bacterial STIs. These strategies have demonstrated efficacy in initial clinical trials in sexual and gender minority communities, but data regarding exacerbation of antibiotic resistance are necessary prior to broad implementation [59, 60, 61]. Our analyses reveal an intricate interplay of behaviours that confer vulnerability to HIV and other STIs, highlighting the need for additional sexual and gender minority‐specific data to better understand vulnerabilities and potential targets for intervention.

Stigma based on gender and sexual practices has garnered increased attention over the last decade, and efforts have intensified to quantify the impact of stigma on health outcomes [62, 63, 64]. Stigma has been associated with increased risk of HIV and decreased psychological health [24, 63, 65, 66]. Our data demonstrate increased stigma in all categories evaluated among TGW as compared to cis‐MSM and non‐binary/other participants. Although high burdens of stigma and depression in TGW population are well documented, there are few other studies that compare TGW to cis‐MSM [62, 67, 68, 69]. Non‐binary/other individuals also generally experienced increased stigma compared to cis‐MSM in our study, though less than the levels observed among TGW. These individuals likely experience augmented stigma than captured in this study due to intersectionality of multiple stigmatized personal facets [70, 71]. Increased outreach and interventions are necessary to reduce stigma and curb the syndemic of stigma and disease acquisition affecting sexual and gender minority populations.

The Nigerian state criminalizes same‐sex sexual behaviour, prohibits gatherings related to same‐sex sexual relationships and imposes penalties for these activities that can include incarceration. Legally sanctioned stigma experienced by sexual and gender minorities negatively affects the health of these individuals [10, 72]. Nigerian leadership has recognized the negative impact of structural stigma by highlighting the importance of stigma reduction as a component of effective HIV reduction in its 2020 consolidated service delivery guidelines. Still, given current legal constraints, public health interventions should focus on mitigation of individual and interpersonal stigma. Community support groups for gender minority populations should be facilitated as this intervention has been previously demonstrated to mitigate the effects of stigma and reduce the negative associated outcomes across multiple body systems [73]. Distributing validated online tools for stigma reduction [74] may provide privacy and accessibility to address individual stigma when the social context limits multilevel interventions.

This study was conducted in urban clinics designed to engage marginalized, vulnerable and diverse sexual and gender minorities, so our findings may not be generalizable to other settings. Study eligibility and recruitment focused on individuals who were identified as male at birth and who participated in sex with men, which resulted in enrolment of some transgender and non‐binary individuals but may have excluded others who did not satisfy inclusion criteria. Gender identity may change over time but was only assessed at study enrolment; future studies should consider repeated longitudinal assessment of gender to explore relationships between gender fluidity and outcomes of interest. Analysis of non‐binary/other participants in aggregate may have obfuscated important differences between individuals with diverse gender and sexual identities, including participants who chose not to categorize their gender; future prospective research should be powered to characterize unique vulnerabilities of more granular gender groups. These analyses were exploratory in nature and each of the identified differences between gender groups merits further independent investigation and robust analysis. The multidimensional dataset available from TRUST/RV368 could be leveraged in future analyses using complex statistical approaches, such as machine learning, to further elucidate relationships between gender identity and STI risk, including the identification of underlying causal mechanisms. Marginalized sexual and gender minorities can be difficult to engage and retain, with previous analyses from our cohort showing 56% loss to follow‐up over the course of the TRUST/RV368 study, potentially biasing results of longitudinal evaluations [75]. Sexual behaviours were assessed via structured interview, which may have been vulnerable to recall and social desirability biases. Many stigma‐related variables were specifically solicited as stigma “due to” sex with men, thus stigma related to other factors, such as membership in a gender minority group or HIV status, may not have been fully captured.

## 5 CONCLUSIONS

Sexual and gender minority populations, including cis‐MSM, TGW and non‐binary/other individuals in Nigeria, have heterogeneous sexual behaviours and diverse vulnerabilities to HIV and other STIs. Of these populations, sexual practices of the non‐binary/other individuals are the least understood and merit specific attention in future studies. The high incidence of HIV and other STIs among TGW and non‐binary/other participants in this study indicates that targeted prevention interventions should be prioritized toward these groups. Study designs that prioritize gender‐specific data gathering, and inclusion of diverse gender minorities, are necessary to inform tailored, rights‐affirming interventions to improve clinical outcomes among sexual and gender minority populations.

## COMPETING INTERESTS

There are no competing interests.

## AUTHORS’ CONTRIBUTIONS

EL analysed the data and authored the first draft of the manuscript. FH and ABM conducted additional data analyses. AK and SA oversaw the collection of clinical data and assisted with the interpretation of these analyses. HQ provided project management support and drafted sections of the manuscript. JAA, MLR, MEC, SDB and RGN designed the TRUST/RV368 study, provided general oversight of the study and assisted with the interpretation of these analyses. TAC coordinated TRUST/RV368 study activities, conceptualized these analyses and oversaw drafting of the manuscript. All authors reviewed this manuscript, provided feedback and approved the manuscript in its final form.

## DISCLAIMER

The views expressed are those of the authors and should not be construed to represent the positions of the U.S. Army, the Department of Defense or the Department of Health and Human Services. The investigators have adhered to the policies for protection of human subjects as prescribed in AR‐70.
